# Mental health among men who have sex with men in Cambodia: Implications for integration of mental health services within HIV programmes

**DOI:** 10.1186/s12939-016-0342-8

**Published:** 2016-03-24

**Authors:** Siyan Yi, Sovannary Tuot, Pheak Chhoun, Khuondyla Pal, Sok Chamreun Choub, Gitau Mburu

**Affiliations:** Research Department, KHANA, Phnom Penh, Cambodia; Programs Department, KHANA, Phnom Penh, Cambodia; Program Impact Unit, International HIV/AIDS Alliance, Brighton, UK; Department of Health Research, Lancaster University, Lancaster, UK

## Abstract

**Background:**

Poor mental health contributes to poor HIV prevention, treatment and care outcomes. This paper documents factors associated with psychological distress among men who have sex with men (MSM) in Cambodia and discusses potential ways in which routine mental health management could be integrated into HIV services.

**Methods:**

A cross-sectional study was conducted in 2014 among 394 MSM randomly selected from two provinces using a two-stage cluster sampling method. A structured questionnaire was used to assess psychological distress, sexual behaviors, substance use, adverse childhood experiences and family dysfunction. Multivariate logistic regression analysis was performed to explore factors associated with levels of psychological distress.

**Results:**

In total, 10.7 % of the respondents reported having suicidal thoughts and 6.6 % reported having attempted to commit suicide in the past three months, while 38.8 % had a higher level of psychological distress (GHQ-12 > 3), which indicates poor mental health. Higher levels of psychological distress were independently associated with older age (AOR = 1.09, 95 % CI 1.03–1.14), alcohol use (AOR = 3.3, 95 % CI 1.36–7.83), illicit drug use (AOR = 3.53, 95 % CI 1.12–11.18), poor self-reported quality of life (AOR = 7.45, 95 % CI 1.79–3.04), and reduced condom use at last sex (AOR = 0.40, 95 % CI 0.21–0.73). MSM with higher levels of psychological distress were significantly more likely to report that a family member said hurtful things to them (AOR = 1.80, 95 % CI 1.10–2.97), a parent or guardian had been physically abused (AOR = 3.51, 95 % CI 1.86–6.62), and a family member had been mentally ill (AOR = 4.01, 95 % CI 2.06–7.81) when they were growing up.

**Conclusions:**

In order to mitigate psychological distress among MSM in Cambodia, integration of mental health interventions within HIV programmes should be strengthened. To achieve optimal impact, these interventions should also address alcohol and other substance use, and low condom use among distressed MSM. In addition, training of clinical and non-clinical HIV service providers to screen for mental health symptoms, and subsequent provision of peer-based outreach and social support for MSM identified with psychological distress is required.

## Background

Poor mental health is a source of significant public health burden globally [1, 2]. In Cambodia, exploration of mental illnesses is particularly relevant given the local history of mass violence, trauma, and genocide, which could also have an impact on mental health of the general population [3]. Available data suggest a background of high levels of psychiatric symptoms among older populations exposed to past traumatic events in Cambodia [4]. Apart from previous exposure to traumatic events, other social factors, including sexual exploitation, family violence, child abuse, trafficking, gambling, and alcohol have been associated with poor mental health in Cambodia [5].

In this context, sexual minorities, including men who have sex with men (MSM), are highly stigmatised based on their sexual orientation [6], and might be at higher risk of mental health issues, depression, and increased suicidal ideation, although comprehensive data are lacking [6]. In Cambodia, HIV is concentrated among MSM, female entertainment workers and injecting drug users. HIV prevalence among these populations is 2.2, 9.8 and 24.8 %, respectively [7–9]. As a result of high levels of HIV prevalence among these groups, they are a key focus for HIV prevention and treatment services. However, there has been very limited provision of mental health services for MSM and other key populations within HIV programmes and indeed, wider health systems in Cambodia [6].

Yet mental health is an important determinant of HIV outcomes: among people living with HIV (PLHIV), untreated or poor mental health contributes to late recruitment onto antiretroviral therapy (ART) [10, 11], poor adherence to ART [12–14] and low retention in care [15, 16]. Among PLHIV, mental disorders have also been associated with higher rates of HIV disease progression [17, 18], hospitalizations [19, 20], viralogical failure [18], and mortality, including suicides [21]. In addition, among HIV-negative individuals, depression and other mental health problems can increase uptake of sexual and other behaviors that increase HIV risk [22], thereby compromising the impact of HIV prevention programmes [23]. In Cambodia, this is relevant to HIV programmes that work with MSM and other populations that are already at high risk of HIV.

Given the increasing evidence of the link between HIV and mental health, it is important to identify ways in which HIV services can be adjusted to adequately address mental health needs of people at risk of or living with HIV [24]. Leveraging on HIV programmes to provide mental health screening is particularly important in Cambodia given the relatively limited mental health services countrywide. National strategy and system of mental health care did not exist until 1993 [25]. By 2010, mental health services were available in only 9 out of 24 provinces [26] and at the end of 2012, there were 49 psychiatrists and 45 psychiatric nurses in the entire country [25]. Although 297 physicians and 270 nurses working with the Ministry of Health have been trained in mental health care [25], mental health services are still poorly accessed [25]. Where available, these services are inequitably concentrated in urban areas [25, 27], and are predominantly 'trauma focused', given the previous legacy of war [28]. As a result, there is a need to find ways to extend mental health services to community-based, primary health, and other non-specialised services based on current epidemiological trends [25, 28]. The main challenge is that there are limited etiological data on the causes of mental health morbidity in Cambodia. Besides trauma and mass violence [28], other correlates of mental distress are largely ignored [29].

## Methods

### Study aims and settings

In response to this gap, a study related to mental health of key populations in Cambodia was conducted. This study was conducted as part of an evaluation of a community-based HIV project known as the Sustainable Action Against HIV and AIDS in Communities (SAHACOM), which was implemented by KHANA, the largest national NGO providing community-based HIV prevention, care, and support services in Cambodia [30]. Participants were MSM recruited from Battambang and Siem Reap province between April and May 2014. This study was the basis of a 2015 paper in which we reported a strong relationship between mental health and stigma among PLHIV [31]. Most recently in 2016, we published additional study findings showing that sex workers who had been forced to drink alcohol at work and those whose clients refused to use a condom experienced high levels of mental distress [32]. In addition, we reported high prevalence of psychological distress among drug users in this study [33]. In this paper, we focus on the findings related to MSM, and make recommendations regarding how HIV programmes can contribute to early screening and identification of mental health conditions among this population.

### Participant recruitment

We used a two-stage cluster sampling method to select participants. Communes in each province were considered the basic sampling unit. In Cambodia, communes are local administrative divisions consisting of 3–30 villages. In total, 32 communes in Battambang and 22 communes in Siem Reap were covered by the SAHACOM project. We included 25 Battambang communes and 13 Siem Reap communes in this study. Communes with less than 20 MSM were excluded.

Probability proportional-to-size sampling was used to select the required number of MSM from each commune. MSM were randomly selected from geographical venues and locations identified as hotspots for HIV acquisition, and preliminarily screened to identify eligible MSM using a screening questionnaire by community health workers. To be included in the study, potential participants needed to be: (1) self-reported as an MSM; (2) at least 18 years of age; (3) available for a face-to-face interview on the day of the data collection; and (4) willing to consent to participate in the study.

### Questionnaire development and training

A structured questionnaire was initially developed in English and then translated to Khmer, the national language of Cambodia. The Khmer questionnaire was then back-translated and pre-tested with a sample of 10 MSM in Phnom Penh to ensure that the intended meanings were retained. Experts working on HIV in key populations in Cambodia also reviewed the questionnaire, which was then revised based on their feedback. The research team members were trained for three days on the study protocol, questionnaire, interview techniques, privacy confidentiality, and quality control strategies, such as rechecking and reviewing the filled questionnaires.

### Variables and measures

To develop the questionnaire, we adapted standardized tools from a previous study in the same population [34], the most recent Cambodia Demographic and Health Survey [35], as well as from previous studies in Cambodia [36]. For socio-demographic characteristics, we collected information on age, marital status, education, occupation, and monthly income. In addition, we collected information on sexual identity, perceived HIV risk, self-rated health and quality of life, and suicidal thoughts and attempts.

The next section collected information on sexual behaviors and diagnosis of HIV and STIs. Condom use was assessed using yes/no questions asking whether participants used a condom at last sex with different types of partners. Yes/no questions were also used to collect information on several other sexual behaviors in the past three months with the above-mentioned partners. We also collected data on HIV testing in the past six months and STI diagnosis in the past three months. To measure substance use, participants were questioned about whether they used any alcohol in the past three months, smoked at least 100 cigarettes in their lifetime, or used any kinds of illicit drugs in the past three months. They were also asked to report the average number of days they got drunk, the average number of cigarettes they smoked per day, and types of illicit drugs they used in the past three months.

Adverse childhood experiences (ACEs) were measured using five questions adapted from the brief screening version of the Childhood Trauma Questionnaire [37, 38]. The questions collected information on the experiences of physical abuse, emotional abuse, sexual abuse, physical neglect, and emotional neglect with five response options ranging from (1) ‘never’ to (5) ‘very often.’ Participants who responded ‘never’ or ‘rarely’ were grouped together as those without ACEs, and those who answered ‘sometimes,’ ‘often,’ or ‘very often’ as those with ACEs. Five items were also adapted from the brief screening version of the Childhood Trauma Questionnaire to enquire about family dysfunction [37, 38]. The items collected information on ‘witnessing violence against a family member,’ ‘having an alcoholic or drug user family member,’ ‘having a family member who was depressed, mentally ill, or who has attempted suicide,’ ‘having parents who had been separated or divorced,’ and ‘having a family member who has been to prison.’ The response options for all the items were ‘yes’ or ‘no,’ except for ‘having parents who had been separated or divorced.’ For this item, another response option was added to indicate if one or both parents had died. In the analysis, participants whose parents had divorced or separated were grouped together with participants whose parent(s) had died.

The final section of the questionnaire assessed psychological distress using a short version of the General Health Questionnaire (GHQ-12) [39]. Each item was rated on a four-point Likert-like scale ranging from “0 = less than usual” to “3 = much more than usual.” The scoring method ‘0-0-1-1’ has been suggested as it is believed to help eliminate biases resulted from respondents who tend to choose responses 0 and 3 or 1 and 2 [40]. The mean score for the whole study population was used as the cut-off to define lower (GHQ-12 ≤ 3) and higher (GHQ-12 > 3) levels of psychological distress as it provides a rough guide to the best threshold [40]. Cronbach’s alpha among MSM in this study was 0.78.

### Data analyses

EpiData version 3 (Odense, Denmark) was used for double data entry. *χ*^2^ test, (or Fisher’s exact test when sample sizes were small) and Student’s *t*-test were used to compare socio-demographic characteristics, self-rated overall health and quality of life, self-perception of HIV risk, substance use, and sexual behaviors among MSM who had a lower level of psychological distress (GHQ-12 ≤ 3) with those among MSM who had a higher level of psychological distress (GHQ-12 > 3). To control for the effects of potential confounding factors, a multivariable logistic regression model was constructed. First, all variables associated with significant levels of psychological distress in bivariate analyses at a level of *p* < 0.2 were simultaneously included in the model. We then removed variables with a *p*-value > 0.05 from the model and the model was refitted. We repeated the steps until all *p*-values of the remaining variables were <0.05 in the final model. Adjusted odds ratio (AOR) were obtained and presented with 95 % confidence intervals (CI) and *p*-values. SPSS version 22 (IBM Corporation, New York, USA) was used for all statistical analyses.

### Ethical statement

Written informed consent was obtained from each participant after they were made clear that participation in this study was voluntary, and they could refuse or discontinue their participation at any time. We protected privacy of the respondents by conducting the interviews at a private place and we did not collect any personal identifiers in the questionnaires or field notes. This study was approved by the National Ethics Committee for Health Research of the Ministry of Health, Cambodia (Reference no. 082NECHR).

## Results

### Socio-demographic characteristics

This study included 394 MSM with a mean age of 23.7 (*SD* = 3.2). The majority of the respondents (90.1 %) were never married and the mean year of their formal education completed was 9.5 (*SD* = 3.2). Over half of the respondents (57.9 %) identified themselves as males and their average income in the past month was US$210 (*SD* = 629). Regarding their self-perception of HIV risk, 35.5 % responded that the level of their HIV risk was higher, while 46.7 % responded that it was lower, than that in the general population. Regarding their mental conditions, 10.7 % reported having suicidal thoughts and 6.6 % reported having attempted to commit suicide in the past three months. Overall, 38.8 % had a higher level of psychological distress (GHQ-12 > 3), which indicates poor mental health.

As shown in Table 1, MSM who had a higher level of mental disorders were significantly older (23.1 ± 4.8 vs. 24.8 ± 5.7, *p* = 0.001) with a lower level of formal education (9.8 ± 3.1 vs. 9.1 ± 3.3, *p* = 0.04) compared to MSM with a lower level of psychological distress. They were significantly more likely to perceive that they were at higher HIV risk compared to the general population (30.3 % vs. 43.8 %, *p* = 0.02) and to rate their overall quality of life as poor or very poor (1.2 % vs. 10.5 %, *p* < 0.001).

**Table 1:** Comparisons of characteristics of MSM with a lower and higher level of psychological distress

Socio-economic characteristics	Total	Total GHQ-12 score		
	(*n* = 394)	≤3 (*n* = 241)	>3 (*n* = 153)	*p*-value^*^
Provinces				0.07
Battambang	329 (83.5)	208 (86.3)	121 (79.1)	
Siem Reap	65 (16.5)	33 (13.7)	32 (20.9)	
Gender identity				0.25
Male	228 (57.9)	139 (57.7)	89 (58.2)	
Female	81 (20.6)	55 (22.8)	26 (17.0)	
Transgender	85 (21.5)	47 (19.5)	38 (24.8)	
Mean age (in year)	23.7 ± 5.2	23.1 ± 4.8	24.8 ± 5.7	0.001
Years of formal education completed	9.5 ± 3.2	9.8 ± 3.1	9.1 ± 3.3	0.04
Marital status				0.91
Never married	355 (90.1)	217 (90.0)	138 (90.2)	
Married and living together	30 (7.6)	19 (7.9)	11 (7.2)	
Divorced/separated/widowed	9 (2.3)	5 (2.1)	4 (2.6)	
Main occupation				0.18
Unemployed	35 (8.9)	17 (7.1)	18 (11.8)	
Students	102 (25.9)	70 (20.9)	32 (20.9)	
Farmer/laborer	59 (15.0)	39 (16.2)	20 (13.1)	
Self-employed	112 (28.4)	66 (27.4)	46 (30.1)	
Other	81 (21.8)	49 (20.3)	37 (24.2)	
Average income in the past month (USD)	210 ± 629	168 ± 505	275 ± 784	0.10
Duration living in current city (in months)	232 ± 106	225 ± 98	244 ± 117	0.09
Self-perception of HIV risk compared to the general population				0.02
Higher	140 (35.5)	73 (30.3)	67 (43.8)	
Same	70 (17.8)	43 (17.8)	27 (17.6)	
Lower	184 (46.7)	125 (51.9)	59 (38.6)	
Self-rated overall health				0.10
Good/very good	147 (37.3)	80 (33.2)	67 (43.8)	
Neither good nor poor	224 (56.9)	147 (61.0)	77 (50.3)	
Poor/very poor	23 (5.8)	14 (5.8)	9 (5.9)	
Self-rated quality of life				<0.001
Good/very good	119 (30.2)	68 (28.2)	51 (33.3)	
Neither good nor poor	256 (65.0)	170 (70.5)	86 (56.2)	
Poor/very poor	19 (4.8)	3 (1.2)	16 (10.5)	
Ever thought to commit suicide	42 (10.7)	23 (9.5)	19 (12.4)	0.37
Ever attempted to commit suicide	26 (6.6)	18 (7.5)	8 (5.2)	0.38

*GHQ* general health questionnaire, *MSM* men who have sex with men

Values are number (%) for categorical variables and mean ± SD for continuous variables

^*^Chi-square test was used for categorical variables and Student’s *t*-test was used for continuous variables

### Substance use

Of total, 88.1 % of the respondents reported having drunk at least a full glass of wine or one can of beer in the past three months. The proportion of respondents who reported having smoked at least 100 cigarettes in their lifetime and used any kinds of illicit drugs in the past three months was 19.5 and 5.1 %, respectively. Table 2 shows that MSM with a higher level of psychological distress were significantly more likely to have drunk at least a full glass of wine or one can of beer (85.5 % vs. 92.2 %, *p* = 0.04) and use any kinds of illicit drugs in the past three months (2.1 % vs. 9.8 %, *p* = 0.001).

**Table 2:** Comparisons of substance use among MSM with a lower and higher level of psychological distress

Substance use in the past 3 months	Total	Total GHQ-12 score		
	(*n* = 394)	≤3 (*n* = 241)	>3 (*n* = 153)	*p*-value^*^
Drank at least a full glass or one can of alcohol	247 (88.1)	206 (85.5)	141 (92.2)	0.04
Mean number of days getting drunk (past month)	5.1 ± 6.7	8.4 ± 9.0	8.2 ± 5.8	0.93
Smoked at least 100 cigarettes in lifetime	77 (19.5)	40 (16.6)	37 (24.2)	0.14
Mean number of cigarettes smoked per day	8.3 ± 7.6	8.4 ± 9.0	8.2 ± 5.8	0.93
Used any kinds of illicit drugs (past three months)	20 (5.1)	5 (2.1)	15 (9.8)	0.001

*GHQ* general health questionnaire, *MSM* men who have sex with men

Values are number (%) for categorical variables and mean ± SD for continuous variables

^*^Chi-square test was used for categorical variables and Student’s *t*-test was used for continuous variables

### Sexual behaviors and HIV/STI testing

As shown in Table 3, risky sexual behaviors of sexually active MSM in this study were common. Many of them were involved in both heterosexual and homosexual relationships, multiple sexual partnerships, and commercial sex; with a mean number of sex partners in the past three months of 3.9 (*SD* = 5.4). In total, 64.0 % had been tested for HIV in the past six months and 7.1 % had been diagnosed with an STI in the past three months. MSM with a higher level of psychological distress were significantly less likely to use a condom at last sex (87.4 % vs. 75.5 %, *p* = 0.003) and to have sex with a girlfriend (65.3 % vs. 50.0 %, *p* = 0.03). However, they were significantly more likely to report having anal sex with boyfriends in the past three months (91.3 % vs. 98.8 %, *p* = 0.03).

**Table 3:** Comparisons of sexual behaviors and HIV/STI testing among MSM with a lower and higher level of psychological distress

Sexual behaviors in the past 3 months	Total	Total GHQ-12 score		
	(*n* = 394)	≤3 (*n* = 241)	>3 (*n* = 153)	*p*-value^*^
Mean number of sex partners	3.9 ± 5.4	3.8 ± 5.7	4.0 ± 5.0	0.68
Used a condom in the last sex	313 (82.8)	202 (87.4)	211 (75.5)	0.003
Had sex with girlfriends	118 (29.9)	79 (32.7)	39 (15.4)	0.03
Mean number of girlfriends you had sex with	1.7 ± 1.1	1.7 ± 1.0	1.9 ± 1.2	0.26
Used a condom in last sex with girlfriends	97 (82.2)	68 (86.1)	29 (74.4)	0.12
Had sex with boyfriends	206 (86.9)	126 (85.7)	80 (88.9)	0.48
Mean number of boyfriends you had sex with	2.4 ± 3.8	2.3 ± 3.5	2.6 ± 4.3	0.53
Used a condom in last sex with boyfriends	192 (92.8)	117 (92.9)	75 (92.6)	0.94
Had anal sex with boyfriends	196 (94.2)	116 (91.3)	80 (98.8)	0.03
Used condom in last anal sex with boyfriend	187 (92.1)	114 (94.2)	73 (89.0)	0.18
Sold sex to men	67 (17.0)	42 (17.4)	25 916.3)	0.78
Used condom when selling sex last time	63 (94.0)	40 (95.2)	23 (92.0)	0.59
Tested for HIV in the past 6 months	252 (64.0)	160 (66.4)	92 (60.1)	0.21
Been diagnosed with an STI	28 (7.1)	16 (6.6)	12 (7.9)	0.63

*GHQ* general health questionnaire, *MSM* men who have sex with men, *STI* sexually transmitted infection

Values are number (%) for categorical variables and mean ± SD for continuous variables

^*^Chi-square test or Fisher’s exact test was used as appropriate for categorical variables and Student’s *t*-test was used for continuous variables

### Adverse childhood experiences and family dysfunction

Table 4 shows that exposure to ACEs were common: 22.8 % of the respondents reported having been physically hurt and 34.0 % reported that a family member said hurtful or insulting things to them when they was growing up. Regarding family dysfunction, 17.8 % reported that their parent or guardian had been physically abused and 29.2 % reported that at least a member of their family had been a problem drinker or drug user. MSM with a higher level of psychological distress were significantly more likely to report that they had been physically hurt (17.4 % vs. 31.4 %, *p* = 0.001), a family member said hurtful or insulting things to them (26.6 % vs. 45.8 %, *p* < 0.001), and someone touched them in a sexual way (10.0 % vs. 20.3 %, *p* = 0.004). They were significantly less likely to report that someone in family made them feel that they were loved (97.5 % vs. 92.8 %, *p* = 0.03). For family dysfunction, MSM with a higher level of psychological distress were significantly more likely to report that their parent or guardian had been physically abused by another family member (10.0 % vs. 30.1 %, *p* < 0.001) and that a family member had been depressed or mentally ill (7.5 % vs. 31.4 %, *p* < 0.001).

**Table 4:** Comparisons of adverse childhood experiences and family dysfunction among MSM with a lower and higher level of psychological distress

Adverse childhood experiences and family dysfunction	Total	Total GHQ-12 score		
	(*n* = 394)	≤3 (*n* = 241)	>3 (*n* = 153)	*p*-value^*^
Adverse childhood experiences (ACEs)				
Physically hurt that needed medical care	90 (22.8)	42 (17.4)	48 (31.4)	0.001
Family member said hurtful or insulting things to me	134 (34.0)	64 (26.6)	70 (45.8)	<0.001
Someone touched me in a sexual way	55 (14.0)	24 (10.0)	31 (20.3)	0.004
Had someone to take care of or protected me	376 (95.4)	232 (96.3)	144 (94.1)	0.32
Someone in family made me feel that I was loved	377 (95.7)	235 (97.5)	142 (92.8)	0.03
Family dysfunction				
Parent or guardian had been physically abused	70 (17.8)	24 (10.0)	46 (30.1)	<0.001
Family member had a drinking problem/drug user	115 (29.2)	64 (26.6)	51 (33.3)	0.15
Family member had been depressed/mentally ill	66 (16.8)	18 (7.5)	48 (31.4)	<0.001
Parents ever been separated or divorced	54 (35.3)	70 (29.0)	54 (35.3)	0.19
Family member had been to prison	12 (3.0)	8 (3.3)	4 (2.6)	0.69

*MSM* men who have sex with men *GHQ* general health questionnaire

Values are number (%)

^*^Chi-square test was used

### Independent factors associated with psychological distress

Results of multivariable logistic regression analysis are shown in Table 5. After adjustment, MSM with a higher level of psychological distress remained significantly more likely to be recruited from Siem Reap (AOR = 2.16, 95 % CI 1.14–4.09), be older (AOR = 1.09, 95 % CI 1.03–1.14), to report poor/very poor overall quality of life (AOR = 7.45, 95 % CI 1.79–3.04), to be an alcohol drinker (AOR = 3.3, 95 % CI 1.36–7.83), and to be an illicit drug user (AOR = 3.53, 95 % CI 1.12–11.18). They were significantly less likely to report using a condom at last sex (AOR = 0.40, 95 % CI 0.21–0.73). Regarding ACEs and family dysfunction, MSM with a higher level mental disorders remained significantly more likely to report that a family member said hurtful or insulting things to them (AOR = 1.80, 95 % CI 1.10–2.97), a parent or guardian had been physically abused (AOR = 3.51, 95 % CI 1.86–6.62), and a family member had been depressed or mentally ill (AOR = 4.01, 95 % CI 2.06–7.81).

**Table 5:** Factors associated with levels of psychological distress among MSM in multivariable logistic regression model

Variables in the final model^a^	Total score of GHQ-12 (≤3 vs. >3)	
	AOR (95 % CI)	*p*-value
Province		
Battambang	Reference	
Siem Reap	2.16 (1.14–4.09)	0.02
Age	1.09 (1.03–1.14)	0.001
Self-rated quality of life		
Good/very good	Reference	
Neither good nor poor	0.84 (0.50–1.41)	0.51
Poor/very poor	7.45 (1.79–3.04)	0.006
Had drunk at least a full glass of alcohol		
No	Reference	
Yes	3.3 (1.36–7.83)	0.008
Used any kinds of illicit drugs		
No	Reference	
Yes	3.53 (1.12–11.18)	0.03
Condom use at last sexual intercourse		
No	Reference	
Yes	0.40 (0.21–0.73)	0.003
Family member said hurtful or insulting things to		
No	Reference	
Yes	1.80 (1.10–2.97)	0.02
Parent or guardian had been physically abused		
No	Reference	
Yes	3.51 (1.86–6.62)	<0.001
Family member had been depressed/mentally ill		
No	Reference	
Yes	4.01 (2.06–7.81)	<0.001

*AOR* adjusted odds ratio, *CI* confidence interval, *MSM* men who have sex with men

^a^Variables associated with psychological distress in bivariate analyses at a level of *p* < 0.2 were simultaneously included in the model, and then variables with a *p*-value ≥0.05 were removed for model fitting, and the steps were repeated until all *p*-values of the remaining variables were <0.05

## Discussion

Mental health is influenced by a range of environmental, social, and individual factors, some of which may be accentuated by the contexts that people live in. Cambodia has a history of trauma and genocide, and generally high levels of psychological distress among its population [3]. However, in this context, daily stressors may be equally or more important causes of current psychological stress than a history of trauma [29]. We believe our study is the first to explore issues of mental health among MSM in Cambodia, in response to calls for aetiological and epidemiological research of mental health in Cambodia [25, 28]. The focus on MSM in this paper is particularly important given that psychologically distressed MSM may experience stigma related to their sexual orientation [6], stigma of mental disorders which remains prevalent in Cambodia [25], and if diagnosed with HIV, HIV-related stigma [41, 42]. Together, these factors could hinder their access to appropriate health services.

Our study demonstrated strong association between psychological distress and older age, low condom use, alcohol use, illicit drug use, as well as verbal abuse and family violence. In addition, there was a strong link between psychological distress and family history of depression and mental illness. These findings are consistent with those from other countries showing strong association of mental disorders with unprotected anal intercourse [43, 44], alcohol and illicit drug use [45], childhood sexual and physical abuse [46], as well as social isolation [47] among MSM. There was also a strong association between psychological distress and cognitive self-awareness of self-rated quality of life in our study, which is consistent with other research findings [48, 49]. Given the high rates of aggressive behaviour in families reported from a recent national survey (11.5 % *n* = 2690) [25], our study emphasises the importance of ACE in development of psychological distress in latter life as reported elsewhere [5, 50]. However, in contrast to other studies [51], we found that older MSMs experienced higher levels of distress. Hypothetically, this may be may be related to possible long term consequences trauma and civil war among older population in Cambodia, which could exacerbate the impact of daily psychological stressors. An alternative hypothesis is that older generation of MSM may experience higher level of stigma related to sexual orientation compared to younger MSM, because it might be more socially acceptable to be MSM among the younger generation in the Cambodian context. These hypotheses would need to be examined in future research.

The negative consequence of poor mental health on HIV prevention, ART adherence, retention in care, and other outcomes is well documented [13, 14, 16–18, 20, 21]. However, evidence suggests that when poor mental health is promptly attended and managed, there are no differences in important HIV outcomes, such as ART initiation and adherence between individuals on mental health treatment and people without mental health conditions [11, 52]. Given these observations, integration of mental health services into HIV prevention, treatment, and care is necessary in order to mitigate the consequences of unattended mental health on the HIV epidemic. Research from other contexts indicate that a significant number of both HIV-positive and negative MSM have mental concerns that are significant enough to require treatment [53], and that generally among PLHIV, these concerns get worse with age [54].

Yet, two recent reviews showed that despite advances in our understanding of the negative impact of mental health on HIV prevention, treatment and care cascade, there is a lack of evidence-based psychosocial interventions that integrate mental health with HIV services [21, 55]. There is a need to develop models of integrating mental health care into HIV services especially given recent evidence showing that poor mental health is the fourth most important cause of hospitalization among PLHIV [19]. We suggest that to achieve optimal integration of mental health and HIV services, a number of health systems-based elements need strengthening.

To begin with, equipping general practitioners, nurses and non-clinical health professionals working in HIV programmes and clinics to screen and identify non-severe mental disorders early, while referring more complex cases to specialised health providers, is required. Expanding competency based training and mentorship related to mental health to staff in HIV organisations would have a significant impact on the ability of the health system to identify early signs of mental illnesses among MSM and other populations who are constantly reached by HIV programmes. In Ethiopia, this was achieved by creating a referral network between generalists in HIV programmes and mental health specialists at mental health facilities [56]. Although mental health training occurs in medical schools [25, 27], there is a lack of mental health training opportunities for staff working in HIV organisations and programmes. Evidence suggests that although staff working in HIV programmes might be willing to perform mental health screening, most have poor knowledge of mental health and often miss opportunities to identify mild forms of psychological distress [57].

In Cambodia, non-governmental organisations provide a significant proportion of HIV services at the community level. Engaging these organisations in routine screening of mental health symptoms could facilitate decentralisation of mental health services to communities. Decentralisation of services has been recommended as a potential strategy to address mental health illnesses in Cambodia [5, 25, 28]. Mental heath screening within HIV programmes would also operationalise task-shifting of basic mental health services to non-clinicians who work in these organisations, such as community health workers, peer-outreach workers, and lay counsellors. In practice, the frequency of mental health screening might vary depending on the available human resources in HIV organisations, as is the case elsewhere [58].

Finally, significant shifts in the organisation of HIV service provision should occur. Successful integration of mental health with HIV services requires changes in organisational structures, infrastructure, and service delivery packages [59]. In Cambodia, HIV services need to be reconfigured to enable the inclusion of peer-based social support for MSM who have been screened and identified as having psychological distress. Social support and outreach can counter mental health morbidity by reducing perceived stigma and social exclusion [60, 61], and enhancing emotional wellbeing and mental health literacy [62, 63]. Engaging HIV programmes in extending the reach of mental health screening could reduce inequality of access to mental health services, given that current access is geographically skewed and disadvantages marginalised groups, including sexual minorities [27], while HIV programmes have national coverage, and predominantly work with sexual minorities, including MSM.

## Conclusions

This study found that psychological distress among MSM in Cambodia is associated with low condom use, alcohol and illicit drug use, older age, poor quality of life, as well as social and family contexts related to child abuse, family violence, and family history of mental illness. However, before firm conclusions can be made regarding our findings, limitations of this study, including limited representativeness of the sample, limited generalizability of the results, and potential recall bias, should be noted. Data used for this study were collected as part of an impact evaluation of SAHACOM, a comprehensive community-based project aiming to improve sexual and reproductive health and quality of life of key populations, including MSM. The levels of health risk behaviours and psychological distress reported in this study may therefore represent a more optimistic picture than in other areas of Cambodia. Moreover, the sampling method was not necessarily designed for this cross-sectional analysis, and adjusting for the sampling effect was difficult. Despite these limitations, findings from this study have important implications for integration of HIV and mental health interventions for MSM in resource-poor settings. Integration of mental health and HIV care has potential to improve HIV care cascade, mitigate perceived HIV stigma and support decentralisation of mental health screening services to MSM and other communities in Cambodia. To achieve optimal impact, models of screening of mental health by staff in HIV service programmes, training of clinical and non-clinical HIV service providers with low knowledge of mental health symptoms, and inclusion of peer-based outreach and social support for MSM identified with psychological distress mental is required.
